# The impact of microbial colonization on cadmium adsorption by rice husk biochar: microorganism-dependent outcomes in bioretention systems

**DOI:** 10.3389/fmicb.2026.1794830

**Published:** 2026-06-01

**Authors:** Carlos Curi-Zavala, Michell K. Arroyo-Julca, Ricardo Flores-Marquez, Emilee Calero-Rios, Sady García, Richard Solórzano-Acosta

**Affiliations:** 1 Facultad de Agronomía, Universidad Nacional Agraria La Molina (UNALM), Lima, Peru; 2 Dirección de Supervisión y Monitoreo en las Estaciones Experimentales Agrarias, Instituto Nacional de Innovación Agraria (INIA), Lima, Peru

**Keywords:** biochar, bioremediation, bioretention, cadmium, *Chlorella*, *Pseudomonas*, *Trichoderma*

## Abstract

**Introduction:**

The use of microorganisms and biochar, individually, has been proposed as a promising strategy for the bioremediation of heavy metals in contaminated water bodies. However, the impact of their combined application, specifically through the immobilization of microorganisms on biochar, on the efficiency of cadmium (Cd) removal and the physicochemical properties of water has not yet been fully assessed, creating uncertainty regarding their effectiveness in decontamination processes. The objective of this research was to evaluate the effect of the physical adsorption capacity for cadmium on rice husk biochar following activation with strains of *Trichoderma harzianum*, *Chlorella* sp., and *Pseudomonas* sp.

**Methods:**

Vertical flow modules were constructed in a factorial design: bioretention systems (M0: biochar, M1: biochar + *Chlorella* sp., M2: biochar + *Pseudomonas* sp., M3: biochar + *Trichoderma harzianum*) × initial cadmium concentration (C1: 3 mg·L^−1^, C2: 6 mg·L^−1^, C3: 15 mg·L^−1^). After 24 h, the efficiency of Cd removal varied significantly according to the system and the initial concentration.

**Results:**

For water with [Cd] ≤ 6 mg·L^−1^, pure biochar (M0) exhibited the highest removal efficiency (99.17 ± 0.41% at 6 mg·L^−1^). For higher concentrations, M1 showed notably enhanced and stable performance, achieving a removal rate of 99.22 ± 1.06%, surpassing M0 (94.35 ± 6.29%) in stability and effectiveness at high loading. In contrast, M2 and M3 systems exhibited lower efficiencies (75.07 ± 12.73% and 77.79 ± 12.22%, respectively, at 15 mg·L^−1^).

**Discussion:**

The results indicate that the outcome of microbial colonization depends critically on the microorganism used. For M2 and M3, removal rates were inversely proportional to the initial Cd concentration, suggesting early saturation of active sites and/or possible ionic competition for nutrient residues. The scaling up of M1 using conventional fertilizers represents a technically and economically sustainable alternative. For M2 and M3, future works should evaluate the incorporation of post-colonization conditioning stages (e.g., washing, pH adjustment) to mitigate potential ionic competition and restore the adsorptive capacity of the biochar.

## 1 Introduction

Cadmium (Cd) is a non-essential and highly toxic heavy metal that occurs in aquatic ecosystems in multiple forms, including dissolved, sediment-bound, adsorbed, and bioaccumulated fractions (Mahmood et al., 2019). Its presence in aquatic environments can adversely affect water quality and disrupt ecological balance (Kubier et al., 2019; Singh and Agrawal, 2010). Although Cd is naturally released from parent rocks through processes such as volcanic eruptions, wildfires, and marine aerosol emissions (Liu et al., 2015), anthropogenic activities have substantially enhanced its mobilization and accumulation in water bodies (Huaraca-Fernandez et al., 2020). Major anthropogenic sources of Cd contamination include mining operations, metal refining activities, industrial effluents, vehicular emissions, and atmospheric deposition (Fuge, 2013; Kubier et al., 2019).

This contamination represents a significant risk to agricultural systems, as Cd can be transferred from contaminated water to soils and subsequently bioaccumulate in crops, adversely affecting plant growth, yield, and product quality (Singh and Agrawal, 2010). Common symptoms of Cd toxicity in plants include chlorosis, wilting, growth inhibition, and cellular damage or death (Gill et al., 2013). Moreover, the accumulation of Cd in edible plant tissues represents a serious concern for human health due to its potential carcinogenic effects (Khan et al., 2017; Waalkes, 2000). To mitigate these risks, environmental regulations have been established. In Peru, the Environmental Quality Standard (ECA) for water used in vegetable irrigation (Category 3. D1) sets a maximum permissible Cd concentration of 0.01 mg·L^−1^ (DS 004-2017-MINAM, 2017), consistent with international guidelines issued by the FAO, WHO, and EPA (Kadeli, 2012; Orosun et al., 2023). However, meeting this regulatory threshold often requires implementing sustainable, effective technological remediation strategies.

Among available remediation strategies, bioretention or biofiltration has emerged as an efficient, cost-effective, and sustainable approach for the removal of contaminants from water (Chaoua et al., 2019). This process relies on the activity of microorganisms immobilized on a filter medium, which retain or transform pollutants, thereby producing a higher-quality effluent with potential for reuse (De Long and Losordo, 2012; Jahanban-Esfahlan et al., 2020). The efficiency of biofiltration systems is strongly influenced by the stability of the microbial biofilm and the supporting substrate (Chaudhary et al., 2003), as well as by environmental factors such as nutrient input, pH, and oxygen availability (Irga et al., 2017; Wang et al., 2023). A wide range of microorganisms, including fungi, microalgae, and bacteria, are commonly employed due to their high capacity for heavy metal removal (Chang et al., 1997; Jiang et al., 2023). Fungi such as *Trichoderma* and *Aspergillus* can detoxify metals through mechanisms including biosorption, intracellular precipitation, and complexation (Dinakarkumar et al., 2024). Microalgae, owing to cell walls enriched with negatively charged functional groups, are capable of adsorbing up to 90% of dissolved metals (Irga et al., 2017). Similarly, bacteria remove metals through surface-associated functional groups, such as –CH, –SO, C=O, N–H, C–N, phosphate, and sulphate groups, that facilitate metal binding and immobilization (Fathollahi et al., 2021; Xu et al., 2020).

Regarding biofilter substrates, biochar, a carbonaceous material produced through the pyrolysis of organic waste, has attracted considerable attention due to its high adsorption capacity, which is attributed to its porous structure, large specific surface area, and abundance of surface functional groups (Chaoua et al., 2019; Kadeli, 2012). The effectiveness of biochar is governed by multiple interaction mechanisms, including van der Waals forces, hydrogen bonding, electrostatic and hydrophobic interactions, and ion exchange processes, whose relative effectiveness depends on the charge and polarity of the target contaminants (He and Davis, 2011; Janu et al., 2021; Tan et al., 2016). Beyond its well-documented performance in bioretention systems (Premarathna et al., 2023), biochar also serves as an effective support for microbial immobilization, enabling the synergistic integration of physicochemical adsorption and biological processes for enhanced pollutant removal (Li et al., 2022). Nevertheless, a deeper understanding of microorganism–biochar interactions and the environmental and operational conditions governing their stability and performance remains necessary (Wang et al., 2024).

However, in complex treatment systems such as biofilters, the presence of coexisting ions in solution (e.g., K^+^, Na^+^, Ca^2+^, and H^+^) can lead to competition for available adsorption sites, thereby substantially reducing the affinity of biochar for specific heavy metals such as Cd^2+^ (Bolan et al., 2014; Wang et al., 2015). This ionic competition may be further intensified in bioaugmented systems, where nutrients supplied to stimulate microbial growth or metabolites released during microbial activity introduce additional competing ions into the system. Consequently, the immobilization of microorganisms on biochar not only modifies the surface characteristics of the support material but also alters the surrounding ionic environment, potentially interfering with the physicochemical adsorption mechanisms responsible for metal removal.

Therefore, this study aimed to evaluate the extent to which microbial colonization influences the physical adsorption capacity of cadmium by rice husk–derived biochar within bioretention systems. The central hypothesis was that the microorganism-dependent immobilization of *Trichoderma harzianum*, *Chlorella* sp., and *Pseudomonas* sp. on biochar could either compromise or enhance its abiotic adsorption capacity for Cd. These effects would occur through active site occupation, reduced porosity, and ionic competition, or alternatively through complementary biosorption mechanisms and pH stabilization, thereby reflecting the contrasting roles of different microbial groups. Ultimately, this research seeks to provide empirical evidence on the balance between these competing mechanisms, thereby contributing to the design of optimized and sustainable bioretention systems for the treatment of heavy metal–contaminated water.

## 2 Materials and methods

### 2.1 Experimental design

A two-factor factorial experimental design was employed, comprising (i) bioretention system (M0: biochar; M1: biochar + *Chlorella* sp.; M2: biochar + *Pseudomonas* sp.; and M3: biochar + *Trichoderma harzianum*), and (ii) initial cadmium concentration (C1: 3 mg·L^−1^; C2: 6 mg·L^−1^; and C3: 15 mg·L^−1^). Each treatment was conducted in triplicate, resulting in a total of 36 experimental units. The experimental units consisted of cylindrical vertical-flow biofilter modules constructed from transparent acrylic, with an internal diameter of 10 cm and a height of 40 cm. All modules were installed outdoors and shaded using Raschel mesh at the La Molina Experimental Centre of the National Institute of Agrarian Innovation (CELM–INIA).

### 2.2 Physicochemical characteristics of the biochar

Biochar was produced from the pyrolysis of rice husks at 600–700 °C for 50 min using a Top Lit Up Draft oven (Bhadha et al., 2014). A homogeneous mixture was obtained, from which a sample was extracted for characterization at the INIA Soil, Water and Foliar Analysis Laboratory (LABSAF-INIA). The pH and electrical conductivity (EC) were analyzed according to Singh et al. (2017), organic carbon and C/N ratio according to ISO 10694 (1995), total nitrogen according to ISO 13878:1998 (1998), and total metals according to EPA 200.8 (U.S. EPA., 1994).

### 2.3 Microbial immobilization and preparation of the biofilter medium

A homogeneous mixture of rice husk biochar (RHB) was used as the support material for the immobilization of three microorganisms of agricultural relevance: *Trichoderma harzianum*, *Pseudomonas* sp., and *Chlorella* sp. While a specific preliminary resistance assay was not conducted as part of this study, the selection of the microbial strains was strictly based on the demonstrated capacity of these taxa to survive and remain functionally active in Cd-contaminated environments (Cayotopa-Torres et al., 2021; Duque-Granda et al., 2019; Elbagory et al., 2021; Xu et al., 2020; Yaghoubian et al., 2019). The microorganisms were produced at the Santa Ana Agrarian Experimental Station of the National Institute of Agrarian Innovation (INIA) following standard cultivation and preservation protocols, with the *Trichoderma* and *Pseudomonas* strains previously characterized by Solórzano-Acosta and Quispe (2024). Microbial inoculants were prepared in nutrient broth and incubated at 28 °C for 48 h to achieve a concentration of 1 × 10^9^ CFU·mL^−1^. Liquid *T. harzianum* inoculum was applied to biochar at a 1:40 (v/v) ratio with the addition of autoclaved molasses and water (1:2, v/v) and incubated for 20 days, while *Pseudomonas* sp. was inoculated onto 20 L of biochar using autoclaved molasses (1:40, v/v) and similarly incubated for 20 days prior to system assembly. The *Chlorella* sp. strain, provided by Biorrefinerías del Perú S. A. C., was scaled up at CELM–INIA to 20 L at a density of 2.78 × 10^6^ cells·mL^−1^ under a 12:12 h light–dark photoperiod with pneumatic agitation using a Nitrofert Fos nutrient solution (4 g·L^−1^). The microalgae solution was applied to biochar at a 1:1 (v/v) ratio and maintained outdoors and shaded under Raschel mesh for 30 days with twice-daily manual agitation (8 a.m. and 5 p.m.) and periodic replenishment of the microalgal culture (2 L every 3 days). Changes in pH and EC of the inoculated biochar were monitored as indirect indicators of successful microbial immobilization.

### 2.4 Characterization of the biofilter medium

The rice husks (RH) used for biochar production, the resulting RHB, and the different biofilter media (i.e., RHB + *T. harzianum*, RHB + *Pseudomonas* sp., and RHB + *Chlorella* sp.) were analyzed to determine the functional groups present, specific surface area, and cation exchange capacity. These analyses were conducted at the CIQTOBIA Laboratory (Centre for Research in Chemistry, Toxicology and Environmental Biotechnology) of the Universidad Nacional Agraria La Molina, the TecMARA Group Laboratory at the Universidad Nacional de Ingeniería, and the LASPAF Laboratory (Laboratory for the Analysis of Soils, Plants, Water and Fertilizers) of the Universidad Nacional Agraria La Molina.

Functional groups were identified by Fourier Transform Infrared Spectroscopy with Attenuated Total Reflectance (FTIR-ATR) using previously ground and sieved samples (15 μm; ≈1,000 mesh) (Smith, 2011). Spectra were recorded in triplicate with 250 scans per sample to optimize the signal-to-noise ratio (Colthup et al., 2012), in accordance with ISO/IEC 17025:2017 (2017) and standardized analytical protocols (Stuart, 2004). Specific surface area was determined using the Brunauer–Emmett–Teller (BET) method (Brunauer et al., 1938) with a Gemini VII 2390 t analyzer (Micromeritics Instrument Corporation, USA), following prior degassing of 0.0546 g of sample at 150 °C for 4 h, in compliance with ISO 9277:2022 (ISO 9277:2022, 2022). Finally, cation exchange capacity (CEC) was determined using the formaldehyde method on 5 g oven-dried samples, which were saturated with NH₄OAc (1 N) at pH 7.0, washed with ethyl alcohol, and subsequently titrated with formaldehyde (Chapman and Pratt, 1973; Guerrero Lázaro, 2019).

### 2.5 Preparation of cadmium solutions

The water used for the preparation of cadmium-enriched solutions was collected from the Rímac River (Lima, Peru) in July 2024 (11°56′39″S, 76°42′06″W), and total metal concentrations were determined in accordance with EPA Method 200.8 (U.S. EPA., 1994; Supplementary Table S1). This approach provided a natural competitive scenario for Cd removal within a complex aqueous matrix, where major cations (Ca: 69.25 mg L^−1^, Mg: 6.94 mg L^−1^, Na: 17.10 mg L^−1^) could compete for adsorption sites, while other trace metals remained at low interference levels. To simulate critical contamination scenarios, assess the resilience of the bioretention media, and enable the detection of statistically significant differences in removal efficiency over short experimental periods (24 h) (Fan et al., 2024), initial Cd concentrations of 3 mg·L^−1^ (C1), 6 mg·L^−1^ (C2), and 15 mg·L^−1^ (C3) were selected. A 24-h assessment period was selected to ensure comparable measurements, consistent with earlier studies that reported quasi-stabilized behavior for various strains (Bazrafshan et al., 2016; Cheng et al., 2017; Kelany et al., 2023; Rocco et al., 2024). The solutions were prepared by dilution of a certified cadmium standard solution preserved in an acidic medium (1,000 ± 10 μg·mL^−1^; Inorganic Ventures; catalogue no. AACD1; Lot Number: S2-CD705778). The prepared solutions were stored in separate containers until use (Table 1).

**Table 1:** Physicochemical characterization of biochar.

Parameter	Unit	Value
pH		10.26
EC	dS·m^−1^	1.79
Bulk density	g·cm^−3^	0.13
Organic carbon	%	42
Organic matter	%	65.10
Total Nitrogen	%	0.65
P_2_O_5_	%	0.0504
K_2_O	%	0.6840
CaO	%	0.102
MgO	%	0.0596
C/N		73.85

### 2.6 Set-up of the experimental units

**Figure 1:**
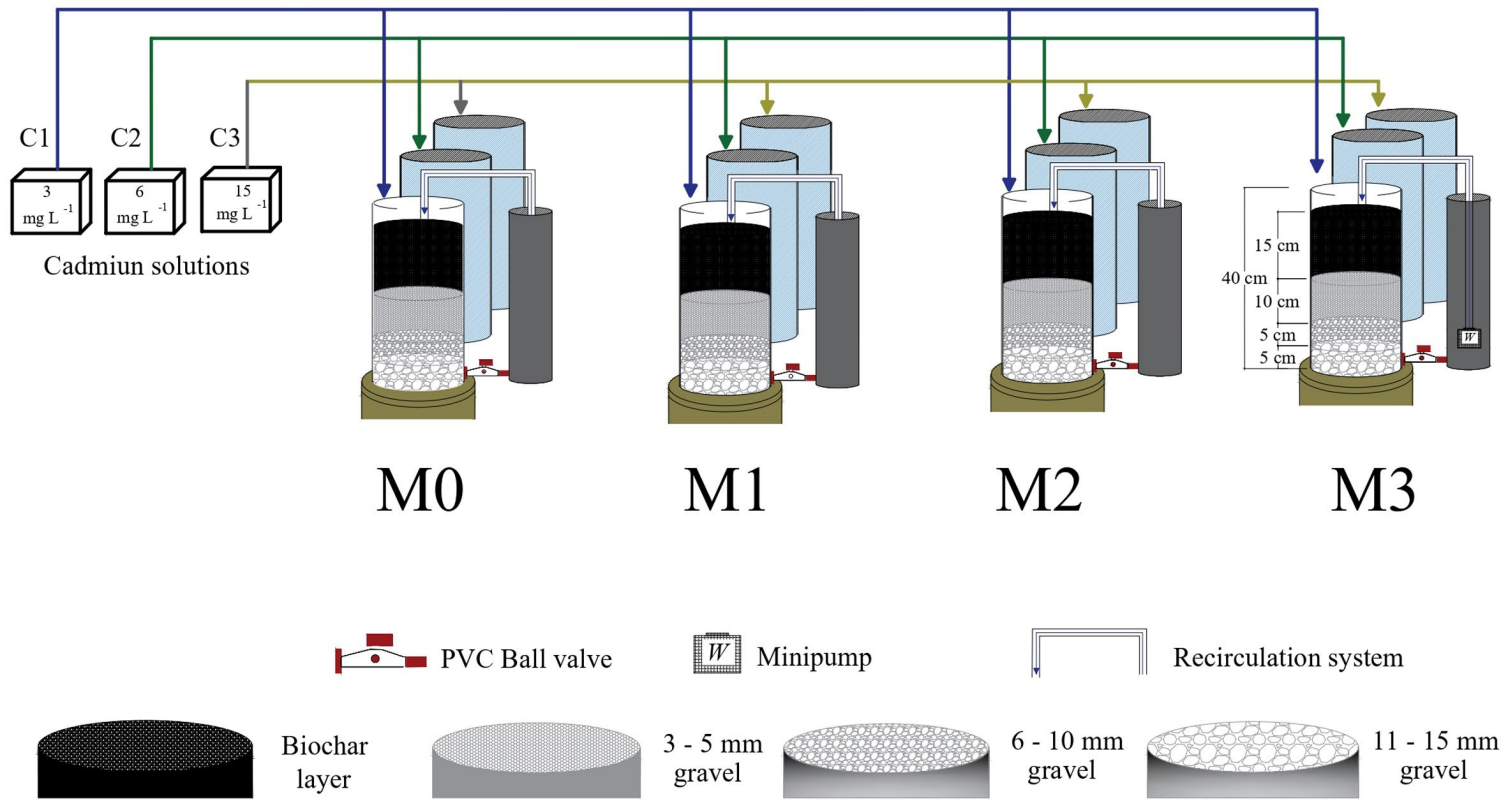
Schematic diagram of the experimental biofilter units.

Within each cylindrical module, a layered biofilter configuration was implemented by assembling four layers with different compositions and heights, as proposed by Chand et al. (2021), depicted in Figure 1. The three lower layers consisted of granular material previously washed with potable water and sieved into three particle size ranges (11–16 mm, 6–10 mm, and 3–5 mm), which were manually placed in ascending order. The upper layer consisted of a homogeneous mixture of washed and sieved fine sand (particle diameter < 0.20 mm) and inoculated biochar at a 1:1 (v/v) ratio, as specified for each treatment. Containers holding the cadmium solutions were positioned 1 m above the top of the modules to generate a positive slope and were connected to the system through a network of polyethylene tubing and control valves, allowing solution distribution according to the experimental design. Each module operated with an independent recirculation system driven by a mini-pump (maximum total dynamic height: 0.6 m; maximum flow rate: 200 L·h^−1^). Following module assembly, recirculation flow rates were measured (0.13 L·h^−1^), and the hydraulic residence time (HRT) was estimated using NaCl as a conservative tracer, following established tracer methodology (Levenspiel, 1999; Wang et al., 2014). The total treated solution volume per experimental unit was 2.7 L.

To isolate the effects of inoculation on the biochar-based bioretention medium, no additional vegetation was included in the mesocosms. Consequently, a modified biosand filter configuration was employed, following the design described by Eniola and Sizirici (2023).

### 2.7 Biofilter operating parameters

Water samples were collected during module filling and from the effluent 24 h after system operation. Inlet samples were obtained at three time points (start, middle, and end of the filling process). Outlet samples were collected by slowly draining the modules using mini-pumps over approximately 30 min. *In situ* measurements of pH, electrical conductivity (EC), temperature (T), total dissolved solids (TDS), and dissolved oxygen (DO) were obtained using a multifunctional water-quality meter (Hanna Instruments, model HI98194). Collected water samples were sent to LABSAF-INIA for laboratory analysis to determine total metal concentrations according to EPA Method 200.8 (U.S. EPA., 1994), nitrogen concentration according to ISO 13878:1998, and phosphorus concentration according to EPA Method 200.8 (U.S. EPA., 1994). The Cd removal efficiency ( $R_{\mathrm{eff}}$ ) in percentage was calculated for each module as:

$$R_{\mathrm{eff}}=\frac{C_{i}-C_{f}}{C_{i}}\times 100$$

where 𝐶_𝑖_ and 𝐶_𝑓_ are the initial and residual concentrations of the evaluated metal in aqueous solution (mg L^−1^), respectively.

### 2.8 Statistical analysis

Differences among treatments were evaluated using a two-way analysis of variance (ANOVA) (*α* = 0.05) applied to biofilter performance variables. Model assumptions were verified using the Shapiro–Wilk test for normality and Levene’s test for homoscedasticity, implemented with the stats package in R version 4.3.0 (Kartikeya, 2019). For variables showing significant differences, mean comparisons were conducted using the Honest Significant Difference (HSD) test (α = 0.05) via the HSD.test function in the agricolae package for R 4.3.0 (de Mendiburu, 2023). Variables that did not meet the assumptions of normality or homogeneity of variance were analyzed using Aligned Rank Transform ANOVA (ART-ANOVA) and the corresponding contrast tests (ART-C) (Elkin et al., 2021), performed with the ARTool package in R 4.3.0 (Kay et al., 2014).

## 3 Results

### 3.1 Characteristics of bioretention systems

**Figure 2:**
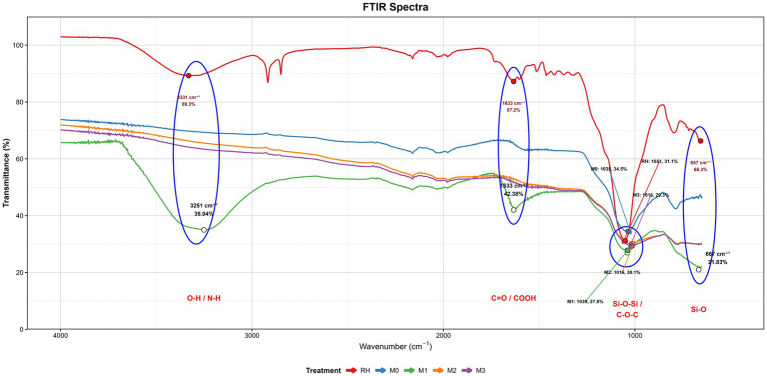
Fourier-transform infrared spectroscopy (FTIR-ATR) spectra of the analyzed materials. RH, Rice husk; M0, biochar; M1, biochar inoculated with *Chlorella* sp.; M2, biochar inoculated with *Pseudomonas* sp.; M3, biochar inoculated with *Trichoderma harzianum*.

Analysis of the FTIR spectra indicated that treatment M1 exhibited the lowest transmittance across the characteristic absorption bands (Figure 2). In the region around 3,251 cm^−1^, associated with O–H and N–H functional groups, M1 showed a transmittance of 35.04%. This trend was also observed at 1633 cm^−1^ (C=O vibrations), 1,039 cm^−1^ (Si–O–Si and C–O–C bonds), and 667 cm^−1^ (Si–O vibrations), where M1 consistently displayed lower transmittance values than treatments M0, M2, and M3. Although differences among the biochar treatments (M0, M1, M2, and M3) were subtle, the transformation from the raw material (RH) to biochar (RHB; M1) was clearly evident.

Biochar production substantially increased the specific surface area, resulting in a balanced distribution between micropores (54% of the total surface area) and mesopores (46%), as shown in Table 2. Microbial inoculation reduced the specific surface area, with a more pronounced effect on microporosity. Consequently, mesopores accounted for approximately 61% of the total surface area in M1 and nearly the entire specific surface area in M2 and M3. The conversion of rice husks into biochar also reduced the cation exchange capacity (CEC) (Table 3), with the non-inoculated biochar (M0; 12.00 cmolc·kg^−1^) exhibiting the highest CEC among the inoculated biochar treatments.

**Table 2:** Distribution of specific surface area between mesopores and micropores.

Media	Specific surface area (m^2^·g^−1^)	Microporosity (m^2^·g^−1^)	Mesoporosity (m^2^·g^−1^)
RH	0.0163 ± 0.0085	0.0206	*
M0	149.4048 ± 3.9144	80.7145	68.6903
M1	108.8709 ± 2.1471	42.6526	66.2183
M2	21.0039 ± 0.5700	*	25.4503
M3	1.4404 ± 0.0553	*	1.4934

*The micropore area is not reported because either the micropore volume is negative or the calculated external surface area is larger than the total surface area.

**Table 3:** Cation exchange capacity of the studied materials.

Bio-filtering media	Code	CEC (cmolc·kg^−1^)
Rice husk	RH	14.40
Rice husk biochar	M0	12.00
Biochar + *Chlorella* sp.	M1	7.20
Biochar + *Pseudomonas* sp.	M2	8.80
Biochar + *Trichoderma harzianum*	M3	8.00

### 3.2 Cadmium removal efficiency

**Figure 3:**
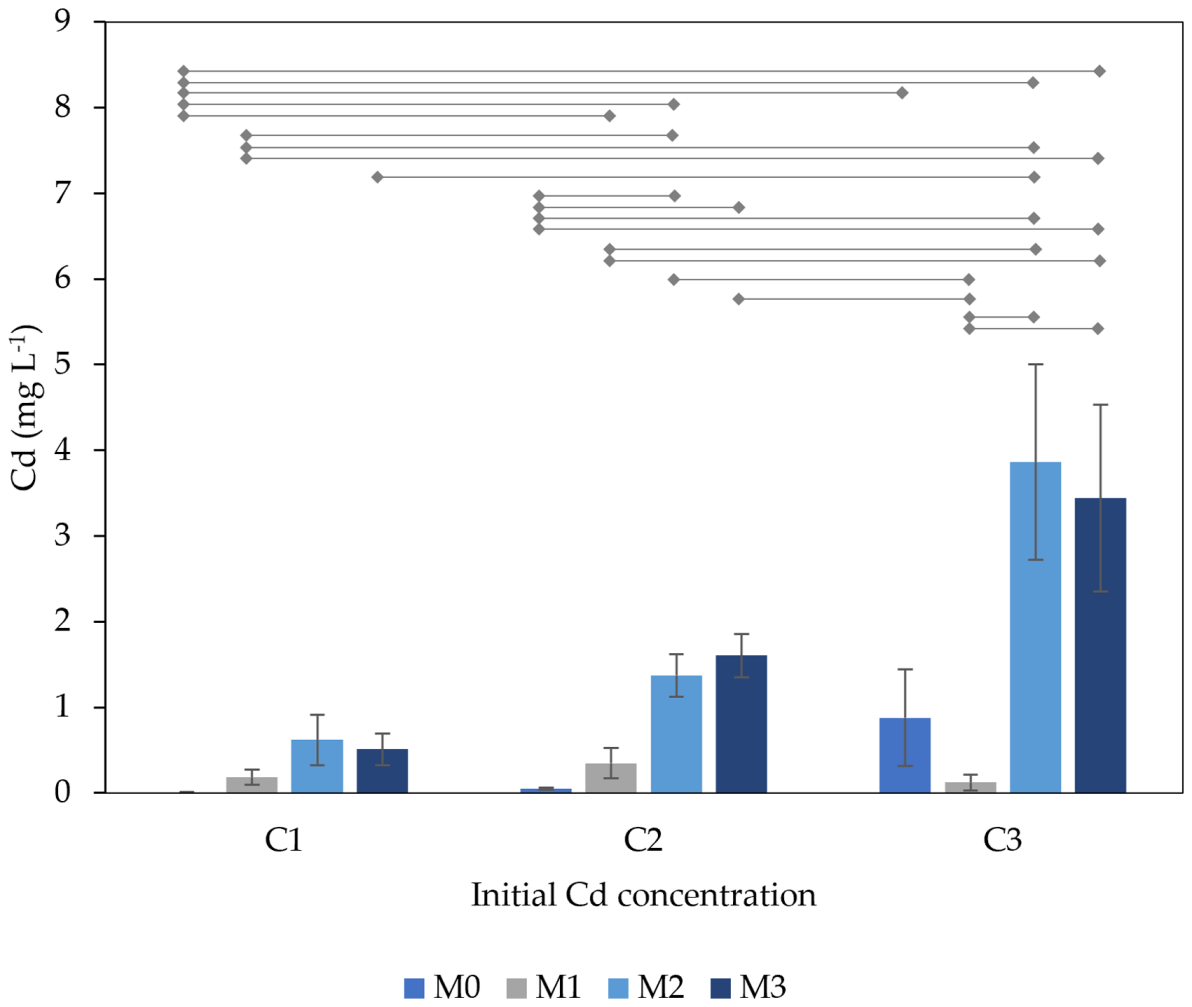
Cadmium (Cd) concentrations in water after treatment. C1, C2, and C3 represent the initial cadmium concentrations. M0, M1, M2, and M3 correspond to the biofiltration layer treatments. Horizontal lines indicate significant differences (*p* < 0.05) according to ART-C contrast tests. Error bars represent the standard error (*n* = 3).

A significant interaction between the two evaluated factors was observed for cadmium (Cd) concentrations in the mesocosm effluents (Table 4). In contrast, Cd removal efficiency (%) was significantly affected only by the main effect of the bioretention system (M). Treatments M0 and M1 achieved mean removal efficiencies exceeding 90%, whereas M2 and M3 achieved approximately 80%. Effluent Cd concentrations from M2 and M3 were directly proportional to the initial Cd concentration (Figure 3). In contrast, M1 showed relatively constant effluent Cd levels, while M0 exhibited an increase in Cd concentration from C3 onward.

**Table 4:** Residual Cd concentrations (mg·L^−1^) and removal efficiencies (%) by treatment.

Treatment		Residual Cd (mg·L^−1^)	Cd removal (%)
C		*	ns
M		*	*
C × M		*	ns
Initial cadmium concentration (C)			
C1		0.32965 ± 0.3689	88.94 ± 12.37
C2		0.84259 ± 0.747	85.98 ± 12.44
C3		2.075 ± 2.086	86.61 ± 13.46
Biochar layer treatment (M)			
M0		0.3111 ± 0.6464	97.75 ± 4.07 ^a^
M1		0.2168 ± 0.2148	95.77 ± 4.49 ^a^
M2		1.9512 ± 1.8017	77.16 ± 11.43 ^b^
M3		1.8511 ± 1.6178	78.01 ± 9.81 ^b^
C × M			
C1	M0	0.0077 ± 0.0024	99.74 ± 0.08
	M1	0.1833 ± 0.1542	93.85 ± 5.17
	M2	0.6183 ± 0.5131	79.26 ± 17.21
	M3	0.5093 ± 0.3168	82.92 ± 10.63
C2	M0	0.0497 ± 0.0248	99.17 ± 0.41
	M1	0.3458 ± 0.3055	94.25 ± 5.08
	M2	1.3725 ± 0.4293	77.16 ± 7.14
	M3	1.6023 ± 0.4404	73.34 ± 7.33
C3	M0	0.8760 ± 0.9753	94.35 ± 6.29
	M1	0.1212 ± 0.1645	99.22 ± 1.06
	M2	3.8629 ± 1.9719	75.07 ± 12.73
	M3	3.4416 ± 1.8939	77.79 ± 12.22

Values are reported as mean ± standard error (*n* = 3). Variables analysed using ART-ANOVA are shaded in grey. ns indicates not significant (*p* > 0.05). *Implies statistical significance at 5%. Means with different lowercase letters (a, b) indicate statistically significant differences among treatments at *p* < 0.05 for the HSD test.

### 3.3 Physicochemical characteristics of the water

After the immobilization period, the biochar–microorganism mixtures exhibited the following pH and EC values: M1, 8.71 and 1,809 μS·cm^−1^; M2, 6.97 and 3,650 μS·cm^−1^; and M3, 7.74 and 5,830 μS·cm^−1^. The hydraulic residence time (HRT) was estimated at 10.3 h. The initial solutions showed the following pH, EC, and dissolved oxygen (DO) values: C1, 6.57, 644.5 μS·cm^−1^, and 3.35 mg·L^−1^; C2, 6.07, 669 μS·cm^−1^, and 3.77 mg·L^−1^; and C3, 2.66, 1205.5 μS·cm^−1^, and 4.46 mg·L^−1^. Nitrogen, phosphorus, and potassium (N/P/K) concentrations were 0.021/0.36/4.14 mg·L^−1^ for C1, 0.01/0.35/4.46 mg·L^−1^ for C2, and 0.01/0.37/4.46 mg·L^−1^ for C3.

The electrical conductivity (EC) of the effluent from M0 was proportional to the initial cadmium concentration (C), as shown in Table 5. The EC values observed for M1 were consistently lower than those of M2 and M3. Higher initial C levels were associated with lower effluent pH, with a marked tendency toward acidic conditions in effluents from M2 and M3. Across all treatments, M1 exhibited the highest dissolved oxygen (DO) concentrations, exceeding the inlet values. In contrast, M0 consistently showed the lowest DO levels, which remained below the inlet concentrations.

**Table 5:** Physicochemical parameters of water following treatment.

Treatments		EC (μS·cm^−1^)	pH	DO (mg·L^−1^)	TDS (mg·L^−1^)
C		*	*	*	*
M		*	*	*	*
C × M		*	ns	ns	*
Initial cadmium concentration (C)					
C1		1783.4 ± 650.9 ^b^	7.112 ± 0.977 ^a^	3.856 ± 0.933 ^a^	877.4 ± 328.37 ^b^
C2		1703.9 ± 534.3 ^b^	7.103 ± 0.833 ^a^	3.590 ± 0.768 ^b^	841.0 ± 265.88 ^b^
C3		2017.9 ± 623.0 ^a^	6.867 ± 0.866 ^b^	3.746 ± 0.870 ^ab^	991.2 ± 314.59 ^a^
Bioretention system (M)					
M0		1183.8 ± 241.3 ^d^	7.770 ± 0.304 ^a^	2.401 ± 0.034 ^d^	592.1 ± 120.74 ^d^
M1		1493.2 ± 217.3 ^c^	7.762 ± 0.145 ^a^	4.484 ± 0.227 ^a^	704.4 ± 80.05 ^c^
M2		2531.7 ± 423.7 ^a^	5.739 ± 0.273 ^c^	4.196 ± 0.371 ^b^	1261.2 ± 212.10 ^a^
M3		2131.7 ± 224.2 ^b^	6.838 ± 0.182 ^b^	3.841 ± 0.161 ^c^	1055.0 ± 102.60 ^b^
C × M					
C1	M0	963.0 ± 78.0 ^h^	7.923 ± 0.150	2.420 ± 0.017	481.7 ± 38.69 ^f^
	M1	1584.7 ± 274.0 ^defg^	7.880 ± 0.202	4.627 ± 0.159	737.7 ± 104.40 ^de^
	M2	2620.3 ± 60.5 ^ab^	5.687 ± 0.470	4.447 ± 0.410	1310.3 ± 30.44 ^ab^
	M3	1965.7 ± 276.5 ^cdef^	6.957 ± 0.103	3.930 ± 0.275	980.0 ± 136.28 ^cd^
C2	M0	1106.3 ± 61.4 ^gh^	7.923 ± 0.150	2.380 ± 0.052	553.3 ± 30.86 ^ef^
	M1	1372.0 ± 87.3 ^gh^	7.690 ± 0.104	4.297 ± 0.197	656.3 ± 13.32 ^ef^
	M2	2069.3 ± 408.5 ^bcde^	5.903 ± 0.060	3.897 ± 0.222	1020.7 ± 179.60 ^c^
	M3	2268.0 ± 114.6 ^bc^	6.897 ± 0.180	3.787 ± 0.081	1133.7 ± 57.05 ^bc^
C3	M0	1482.0 ± 87.3 ^fgh^	7.463 ± 0.335	2.403 ± 0.021	741.3 ± 43.66 ^de^
	M1	1523.0 ± 265.1 ^efgh^	7.717 ± 0.023	4.530 ± 0.236	719.3 ± 95.36 ^ef^
	M2	2905.3 ± 75.0 ^a^	5.627 ± 0.093	4.243 ± 0.319	1452.7 ± 37.63 ^a^
	M3	2161.3 ± 202.2 ^bcd^	6.660 ± 0.123	3.807 ± 0.061	1051.3 ± 50.64 ^c^

*Implies statistical significance at 5%. Values are reported as mean ± standard error (*n* = 3). Means with different lowercase letters (a, b, c, d, e, f, g, h) indicate statistically significant differences among treatments at *p* < 0.05 for the HSD test.

### 3.4 Nutrient consumption

No significant differences in nitrogen (N) concentrations were observed among the effluents from treatments M1, M2, and M3 (Table 6). Phosphorus (P) concentrations were significantly affected only by the main effect of the bioretention system (M), with values for M1 markedly higher than those for M2 and M3 (M1 ≫ M2 ≈ M3). Regarding potassium (K), the lowest concentrations were associated with the main effects of C2 and M1, which differed significantly from the remaining treatment levels.

**Table 6:** Concentrations of N, P, and K in the water following the application of the proposed treatments.

Treatments		N (mg·L^−1^)	P (mg·L^−1^)	K (mg·L^−1^)
C		ns	ns	*
M		ns	*	*
C × M		ns	ns	ns
Initial cadmium concentration (C)				
C1		40.839 ± 7.543	13.325 ± 16.576	246.016 ± 166.180 ^ab^
C2		45.586 ± 2.268	12.298 ± 14.725	171.941 ± 119.480 ^b^
C3		42.553 ± 2.085	15.026 ± 17.483	258.633 ± 162.220 ^a^
Biochar layer treatment (M)				
M1		41.972 ± 5.684	34.057 ± 9.341 ^a^	55.4161 ± 20.386 ^b^
M2		41.874 ± 5.618	4.441 ± 0.983 ^b^	350.597 ± 130.570 ^a^
M3		45.133 ± 2.791	2.153 ± 0.845 ^b^	270.577 ± 56.907 ^a^
C × M				
C1	M1	38.940 ± 9.517	33.68 ± 12.62	54.387 ± 7.437
	M2	38.116 ± 9.354	4.627 ± 0.597	416.936 ± 88.501
	M3	45.461 ± 0.959	1.668 ± 0.645	266.724 ± 55.014
C2	M1	45.248 ± 1.139	31.247 ± 7.469	42.699 ± 13.086
	M2	43.850 ± 2.539	3.709 ± 0.948	226.592 ± 118.430
	M3	47.662 ± 1.294	1.9505 ± 0.709	246.532 ± 70.869
C3	M1	41.728 ± 2.719	37.242 ± 10.32	69.162 ± 30.136
	M2	43.655 ± 0.686	4.996 ± 1.126	408.263 ± 108.120
	M3	42.275 ± 2.561	2.8417 ± 0.898	298.474 ± 53.363

Values are reported as mean ± standard error (*n* = 3). Variables analyzed using ART-ANOVA are shaded in grey. ns indicates not significant (*p* > 0.05). *implies statistical significance at 5%. Means with different lowercase letters (a, b) indicate statistically significant differences among treatments at *p* < 0.05 for the HSD test.

The highest Cd removal efficiencies were observed in effluents with higher pH, DO, and P concentrations, along with lower EC, TDS, and K levels. Notably, pH was negatively correlated with EC and K, and positively correlated with P. In addition, higher DO concentrations were positively associated with increased P levels.

## 4 Discussion

Rice husk biochar (RHB) enabled efficient microbial colonization, thereby modifying its physicochemical properties for bioretention applications. Consistent with previous studies (Ippolito et al., 2020; Wang et al., 2018; Wei et al., 2019), our results indicate that the thermal conditions employed during RHB production promoted an increase in specific surface area, particularly microporosity, and the formation of aromatic carbonaceous structures (Liu et al., 2010; Wei et al., 2017). These characteristics are known to enhance the availability of potential adsorption sites for heavy metals (Ahmedna et al., 2004; Beesley et al., 2010; Ghorbani et al., 2019; Qiu et al., 2021; Suksabye et al., 2016; Weber and Quicker, 2018). However, BET analysis revealed a pronounced reduction in porosity for inoculated biochar (M1, M2, and M3) compared with the non-inoculated biochar (M0), indicating successful and extensive microbial colonization that occluded the biochar’s pore structure. This structural change implies that the internal active sites of the biochar, which would typically facilitate physical adsorption, ion exchange and surface complexation (Li et al., 2017), were masked by the microbial biofilm. Similarly, the decrease in specific surface area relative to M0, the lower FTIR transmittance observed in inoculated treatments, and the reduction in cation exchange capacity (CEC) collectively suggest the formation of a biomolecular layer coating on the biochar surface in treatments M1, M2, and M3. This biological interface would alter the surface properties of the biochar, shifting the predominant removal drivers from the mechanisms within the micropore network toward surface-mediated processes on pore occluded network in inoculated treatments. Consequently, the Cd removal observed in the inoculated systems would be attributed to a reactive interface based on the metabolic and biosorptive activity of the biofilm, and to the intrinsic capacity of surface complexation and ion exchange of the biochar, thus maintaining high removal efficiencies. Nevertheless, to quantify the individual contribution of each pathway (i.e., complex interplay of ion exchange, surface complexation with microbial functional groups, and microenvironment-induced precipitation), high-resolution spectroscopic techniques (e.g., X-ray Photoelectron Spectroscopy (XPS) or elemental tracking – CHN) would be required (F. Zhang et al., 2025). Therefore, our attribution of these effects relies on the observed correlation between the loss of physical porosity and the maintenance of removal efficiency, suggesting that biological biosorption compensates for the loss of available physical adsorption sites. Accordingly, the lower dissolved oxygen concentrations observed in M0 may be attributed to favorable conditions for the naturally occurring aerobic microorganisms in the aqueous medium. Recirculation-induced oxygenation of the medium likely enhanced their heterotrophic respiration, resulting in increased DO consumption (Long et al., 2024). In contrast, in systems inoculated with microorganisms, these communities occupied the available niches and became the primary consumers of DO.

The immobilization of *Chlorella* sp. microalgae on biochar resulted in the most stable and complementary system among the evaluated treatments. This superior performance can be attributed to the combined action of three complementary mechanisms. First, passive biosorption was mediated by carboxyl (–COOH) and phosphate (–PO₄) functional groups present in the microalgal cell wall (Monteiro et al., 2011; Muñoz et al., 2006). The presence of these groups is supported by the FTIR bands at 1633 cm^−1^, corresponding to C=O vibrations of carboxylates and amides, and at 1039 cm^−1^, associated with C–O–C and P–O bonds, which exhibited the lowest transmittance in treatment M1. Second, continuous photosynthetic activity contributed to system stability by releasing molecular oxygen (Kazbar et al., 2019), increasing DO concentrations to >4.5 mg·L^−1^, and by stabilizing the pH within a near-neutral range (7.7–7.9). These conditions favored the precipitation of Cd as carbonate and hydroxide species while preserving the functionality of biosorptive groups (Wang et al., 2022). Third, the moderate release of extracellular polymeric substances (EPS) provided additional binding sites for Cd^2+^ ions (Shen et al., 2017) without fully obstructing the biochar pore network, thereby preserving approximately 47% of the original microporosity. This partial preservation of pores likely exerted a buffering effect, reducing microalgal cell lysis under acidic conditions. In contrast to the bacterial and fungal systems, this combination of physicochemical and biological processes enabled M1 to maintain high Cd removal efficiency (99.22 ± 1.06% at 15 mg·L^−1^), even when the purely physical adsorption capacity of pristine biochar (M0) began to exhibit signs of saturation. The detected N, P, and K concentrations are attributed to residual inputs from the immobilization nutrient solution. Moreover, the near-neutral pH enhanced the solubility of phosphorus species (Cho et al., 2011), maintaining their bioavailability while limiting irreversible adsorption. The ability of M1 to neutralize the initial acidity of treatment C3 highlights a proactive buffering capacity driven by the resilience of the bio-composite offering a key advantage for treating real-world fluctuating effluents.

**Figure 4:**
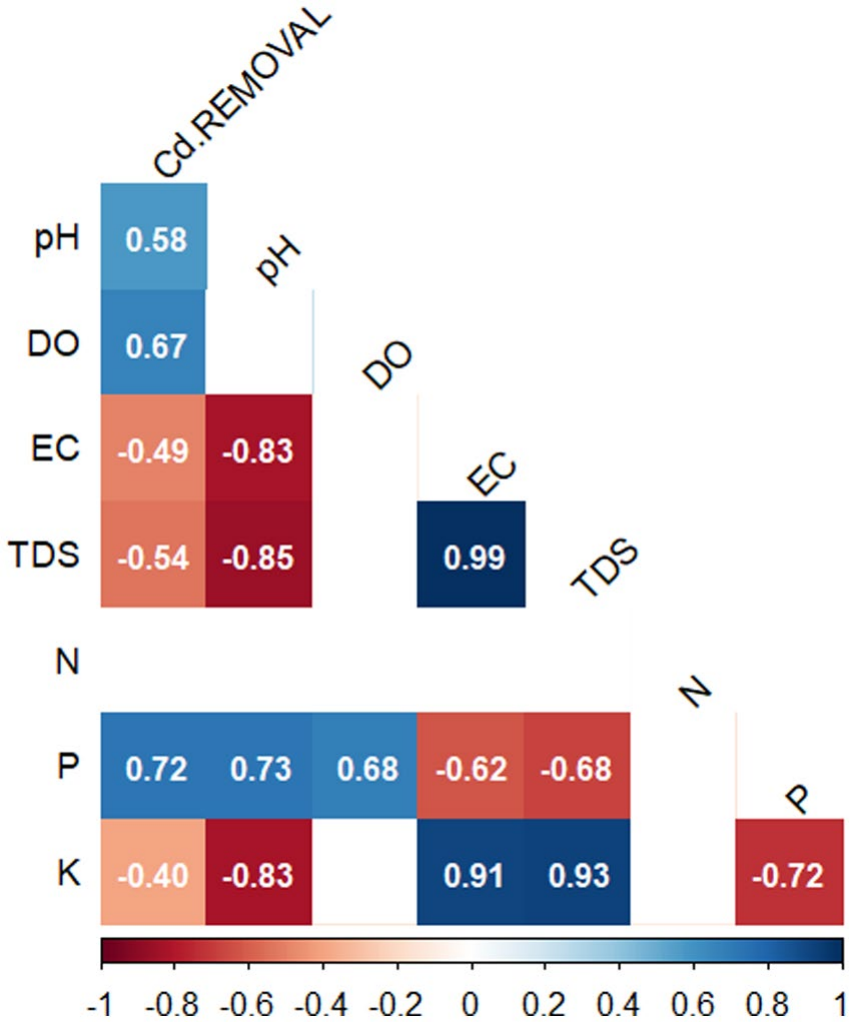
Spearman correlation matrix for Cd removal, physicochemical parameters, and nutrient consumption in mesocosm effluents. Only significant correlation coefficients (*p* < 0.05) are shown.

Inoculation with *Pseudomonas* (M2) and *Trichoderma* (M3) decreased the CEC of the biofilter system. Although these treatments maintained a higher density of negative surface charges than M1, they reduced the availability of exchangeable sites. The observed reduction in porosity, together with higher CEC values relative to M1, suggests that a substantial proportion of the negative charges originated from the inoculated and proliferated biomass rather than from accessible biochar surfaces. The presence of *Pseudomonas* (M2) induced pronounced acidification of the medium (pH ≈ 5.7), likely due to the production and release of organic acids such as gluconic acid or 2-ketogluconate. This response is a well-documented mechanism employed by *Pseudomonas* spp. under metal stress to solubilise and chelate metal ions (Ayangbenro and Babalola, 2017). However, the resulting decrease in pH likely led to protonation of biochar adsorption sites (e.g., –COO^−^ to –COOH), thereby reducing their affinity for Cd^2+^; indeed, protonation of carboxylic and phenolic groups under acidic conditions is known to diminish metal binding capacity on biochar surfaces (Huang et al., 2024). Furthermore, the use of molasses as a carbon substrate introduced a substantial load of residual K^+^ ions (Mordenti et al., 2021), which may have competed with Cd^2+^ for available cation exchange sites, although direct evidence of such competition requires further experimental confirmation. This competitive effect is supported by the strong inverse correlation observed between effluent K^+^ concentrations and Cd removal efficiency (Figure 4). At the cellular level, *Pseudomonas* cell walls, rich in lipopolysaccharides containing phosphate and carboxyl functional groups, can initially adsorb Cd^2+^ ions (Oh et al., 2009). However, bacterial cell wall sulfhydryl sites exhibit high affinity for Cd but comprise only 5–10% of total binding sites and saturate rapidly under high metal loading (Fein et al., 2019). Nevertheless, the adsorption capacity of this biological system appears to have been rapidly saturated, thereby limiting its performance at higher Cd concentrations.

In turn, the extensive hyphal colonization of *Trichoderma harzianum* on biochar (M3) promoted rapid initial surface adsorption, taking advantage of the high density of functional groups (amino, carboxyl, and phosphate) present in its chitin- and glucan-rich cell wall. However, the observed proportional relationship between Cd removal and initial concentration suggests that this mechanism was rapidly saturated. This limitation may be attributed to the fact that intracellular uptake (bioaccumulation) relies on membrane transport systems, such as ZNT or ZIP proteins, which have limited transport capacities and whose expression or activity may have been constrained under the experimental conditions (Cacciola et al., 2015). Subsequent Cd^2+^ chelation by polyphosphates within vacuoles and intracellular compartmentalization are metabolically demanding and comparatively slow processes, which likely explains the lower removal efficiency observed within the short experimental duration (24 h). Moreover, although the filamentous morphology of *T. harzianum* provides a large contact surface, it may also form a physical barrier that limits access to the biochar’s internal pore structure, as evidenced by the pronounced reduction in specific surface area. Notably, while *T. harzianum* can produce metal-chelating metabolites like harzianic acid and adsorb Cd in their cell walls (Tommaso et al., 2021), these mechanisms are also subject to saturation and may not enhance overall Cd removal beyond biochar’s physical adsorption.

While our study focused primarily on Cd removal efficiency and surface adsorption mechanisms, complementary biotransformation pathways and EPS-mediated processes may have also contributed to the observed outcomes, particularly in M1 and M2. Among the genera identified in our sequencing data, *Pseudomonas* (specifically *P. stutzeri* and *P. aeruginosa*) is widely recognized for producing alginate and other extracellular polymeric substances (EPS) containing carboxyl and phosphate groups that effectively chelate Cd^2+^ (Balíková et al., 2022). Furthermore, certain *Pseudomonas* strains possess enzymatic reduction systems (e.g., sulfate reductases) that can precipitate Cd as cadmium sulfide (CdS), representing an active biotransformation pathway beyond passive biosorption. For *Trichoderma harzianum* (M3), the production of harzianic acid and other metal-chelating metabolites has been documented (Tommaso et al., 2021), although its effectiveness under our experimental conditions appeared limited.

In the *Chlorella* sp. system (M1), we propose that Cd-EPS complex formation, followed by subsequent precipitation as carbonate (favored by the near-neutral to alkaline pH generated by photosynthetic activity, 7.7–7.9), represents an active detoxification pathway (Lourembam et al., 2024). This mechanism likely operated alongside passive biosorption, contributing to the enhanced stability and high removal efficiency observed in M1 at elevated Cd concentrations. Conversely, in M2 (*Pseudomonas* sp.), the pronounced acidification of the medium (pH ≈ 5.7) suggests the production of organic acids such as glucuronic acid, which can solubilize Cd (Qian et al., 2022). However, this may have a paradoxical effect by mobilizing Cd^2+^ rather than immobilizing it, potentially explaining the reduced removal efficiency observed in M2 compared to M0 and M1. These observations highlight that microbial colonization can lead to either immobilization or mobilization of Cd depending on the specific metabolic traits of the inoculated microorganism. Future studies integrating metabolomic, proteomic, or transcriptomic analyses would be necessary to fully disentangle the relative contributions of surface adsorption versus active biotransformation pathways in microorganism-biochar composites.

Several limitations were identified that constrained a comprehensive understanding of the effects of microbial inoculation on the bioremediation capacity of biochar in Cd-contaminated aqueous environments. The absence of evaluations at multiple time points limited the assessment of temporal changes in the adsorption and removal capacities of the biofilter media. Future studies incorporating extended and multiple sampling intervals would enable the determination of saturation dynamics and breakthrough times for each treatment. That assessment would allow life-time estimations, especially for field-scaling processes. Even though the use of natural river water enriched would represent a first step into the field-scaling related to the natural multielement competition, complementary studies must be performed to evaluate biochar regeneration options. Chemical regeneration experiences have been reported for heavy metals treatment showing progressive reduction in biochar’s adsorption capacity but within functional levels for practical applications (Alsawy et al., 2022). Complementary, the biochar reinoculation will be necessary to replace microbial communities and preserve the equilibrium between biofilm development and adsorption capacity of the system (Detman-Ignatowska et al., 2026). In that sense, recent studies reported the combined use of biochar within constructed wetlands as an alternative to enhance the treatment of heavy-metal contaminated water. Although better heavy metal remediation rates and enhancement in microbial dynamics have been reported (Ding et al., 2025; Feng et al., 2022, 2024; Zhang et al., 2024); clogging risk and proliferation of undesirable microbial communities are potential tradeoffs (Yu et al., 2023). Thus, the pre-inoculation of beneficial microbiota onto the biochar would prevent part of these potential tradeoffs but must be accompanied by adequate management protocols in which future studies should focus.

On the other hand, the absence of microorganism-only treatments limited the assessment of true synergistic interaction versus the individual contribution of biochar and microorganisms. The observed performance could alternatively be explained by the additive effect of physical adsorption by biochar and biosorption by *Chlorella* sp. Thus, future studies incorporating individual components as controls are required to elucidate each microorganism’s specific contributions and their metal detoxification mechanisms. Additional microscopic evidence (e.g., SEM) and high-resolution spectroscopic techniques (e.g., XPS, CHN) would provide a more comprehensive validation and enhance the differentiation of metal detoxification mechanisms of each treatment. Furthermore, evaluations using water collected from contaminated environments are required. The presence of complex mixtures of highly loaded heavy metals would allow investigation of synergistic and antagonistic interactions among co-occurring contaminants, thereby improving the environmental relevance of the findings. From a microbiological perspective, the viable microbial biomass immobilized on biochar was not quantified before or after Cd exposure. Such measurements would have enabled correlations between cell viability and removal efficiency. Moreover, specific microbial exudates produced under metal stress, such as organic acids, siderophores, and EPS, were not characterized, despite their critical role in metal detoxification mechanisms. Future research should therefore integrate microbiological and analytical techniques, including colony-forming unit (CFU) quantification, scanning electron microscopy (SEM) to visualize spatial colonization, and exometabolomic analyses using liquid chromatography–mass spectrometry (LC–MS), to comprehensively elucidate interactions at the biochar–microorganism–contaminant interface. In addition, while the lack of pH standardization reflects operational reality, we acknowledge that it introduces complex interactions regarding Cd speciation and surface charge. Future mechanistic studies should incorporate high-resolution monitoring of H^+^ flux and Cd^2+^ activity to further decouple the synergistic effects of pH and metal concentration.

Taken together, our findings demonstrate that microbial colonization does not uniformly compromise the Cd adsorption capacity of rice husk biochar. Instead, the outcome is strongly microorganism dependent. The immobilization of microorganisms on biochar constitutes a trade-off, in which part of the intrinsic physical adsorption capacity of biochar may be reduced in exchange for the incorporation of biologically mediated remediation processes through microorganisms. The overall effectiveness of the resulting composite material is therefore governed by the balance between these abiotic and biotic mechanisms. For water remediation scenarios involving Cd concentrations below 6 mg·L^−1^, biofiltration using pristine rice husk biochar (M0) proved to be the most effective approach. In contrast, at higher Cd concentrations, inoculation with *Chlorella* sp. microalgae (M1) enabled a more favorable complementarity between biochar adsorption and biological removal processes, surpassing pristine biochar in stability and effectiveness under high Cd loading. From an applied perspective, system scale-up using readily available fertilizers as nutrient sources represents a technically feasible and economically sustainable option without compromising the heavy metal adsorption capacity of the biochar. On the other hand, the use of bacterial (M2) and fungal (M3) inoculants would require the incorporation of complementary post-colonization treatments (e.g., washing, pH adjustment) to mitigate potential ionic competition and restore the physicochemical adsorption capacity of the biochar (Ding et al., 2021; Bahuguna et al., 2025). In this context, the use of molasses as a low-cost promoter of microbial growth and biofilm formation on biochar offers a promising strategy for scaling up such systems, consistent with circular economy principles and the revalorization of agro-industrial by-products. Furthermore, our results demonstrate the biofilters potential as robust pre-treatment units capable of significantly reducing the primary chemical load before subsequent polishing stages.

## Data Availability

The original contributions presented in the study are included in the article/Supplementary material, further inquiries can be directed to the corresponding author.
