# Supplementary material for: The impact of microbial colonization on cadmium adsorption by rice husk biochar: microorganism-dependent outcomes in bioretention systems
Source: Front Microbiol. 2026 Jun 1;17:1794830. doi: 10.3389/fmicb.2026.1794830 (PMC13265493; doi:10.3389/fmicb.2026.1794830)
Supplement: Supplementary file 1 [file Table_1.docx]

**Supplementary material**

**Table S1.** Characterization of metal analytes in water samples collected from the Rímac River.

| Parameter | Unit | Value |
| --- | --- | --- |
| Aluminum (Al) | mg·L^-1^ | 0,001 |
| Antimony (Sb) | mg·L^-1^ | 0,004 |
| Arsenic (As) | mg·L^-1^ | 0,031 |
| Barium (Ba) | mg·L^-1^ | 0,053 |
| Beryllium (Be) | mg·L^-1^ | 0,001 |
| Boron (B) | mg·L^-1^ | 0,002 |
| Cadmium (Cd) | mg·L^-1^ | 0,001 |
| Calcium (Ca) | mg·L^-1^ | 69,254 |
| Cobalt (Co) | mg·L^-1^ | 0,001 |
| Copper (Cu) | mg·L^-1^ | 0,016 |
| Chromium (Cr) | mg·L^-1^ | 0,005 |
| Strontium (Sr) | mg·L^-1^ | 1,375 |
| Iron (Fe) | mg·L^-1^ | 0,127 |
| Manganese (Mn) | mg·L^-1^ | 0,042 |
| Magnesium (Mg) | mg·L^-1^ | 6,945 |
| Mercury (Hg) | mg·L^-1^ | 0,000 |
| Molybdenum (Mo) | mg·L^-1^ | 0,007 |
| Nickel (Ni) | mg·L^-1^ | 0,007 |
| Potassium (K) | mg·L^-1^ | 3,326 |
| Silver (Ag) | mg·L^-1^ | 0,003 |
| Lead (Pb) | mg·L^-1^ | 0,015 |
| Selenium (Se) | mg·L^-1^ | 0,144 |
| Sodium (Na) | mg·L^-1^ | 17,106 |
| Thallium (Ti) | mg·L^-1^ | 0,000 |
| Vanadium (V) | mg·L^-1^ | 0,011 |
| Zinc (Zn) | mg·L^-1^ | 0,112 |
